# Emergence of Multidrug-Resistant and Biofilm-Producing *Staphylococcus aureus* from Raw Poultry in Algeria: Implications for Public Health

**DOI:** 10.3390/microorganisms13122764

**Published:** 2025-12-04

**Authors:** Feryal Belfihadj, Meriem Elkolli, Naouel Boussoualim, Amira Bourouba, Charefeddine Mouffok, Maryem Kraouia, Vesna Milanovic, Andrea Osimani, Lucia Aquilanti, Mohammad Raish, Byong-Hun Jeon, Hyun-Jo Ahn, Yacine Benguerba

**Affiliations:** 1 Laboratory of Applied Microbiology, Faculty of Natural and Life Sciences, University of Ferhat Abbas Setif 1, Setif 19000, Algeria; belfihadjferyal@gmail.com (F.B.);; 2 Laboratory of Applied Biochemitry, Faculty of Natural and Life Sciences, University of Ferhat Abbas Setif 1, Setif 19000, Algeria; 3 Laboratory of Cellular Toxicology, Badji Mokhtar-Annaba University, P.O. Box 12, Annaba 23000, Algeria; 4 Laboratory of Improvement and Development of Plant and Animal Production, University of Ferhat Abbas Setif 1, Setif 19000, Algeria; 5 Department of Agricultural, Food, and Environmental Sciences (D3A), Università Politecnica delle Marche (UNIVPM), Via Brecce Bianche, 60131 Ancona, Italy; m.kraouia@pm.univpm.it (M.K.);; 6 Department of Pharmaceutics, College of Pharmacy, King Saud University, P.O. Box 2457, Riyadh 11451, Saudi Arabia; 7 Department of Earth Resources and Environmental Engineering, Hanyang University, 222 Wangsimni-ro, Seongdong-gu, Seoul 04763, Republic of Korea; 8 Laboratoire de Biopharmacie et Pharmacotechnie (LBPT), University of Ferhat Abbas Setif 1, Setif 19000, Algeria

**Keywords:** *S. aureus*, raw chicken meat, antibiotic resistance, resistance genes, MDR, biofilm

## Abstract

*Staphylococcus aureus* is a common foodborne pathogen, posing significant concern due to the emergence of its multidrug-resistant (MDR) strains. The aim of this study was to assess the antibiotic resistance profiles in *S. aureus* isolated from raw poultry, the associated resistance genes, and their ability to form biofilms. *S. aureus* was isolated and identified using conventional microbiological methods. Antimicrobial susceptibility profiles were assessed using the disk diffusion method, and biofilm-forming ability was evaluated using the microtiter plate assay. The presence of antimicrobial resistance genes was determined by PCR. A total of 45 isolates were isolated. High resistance rates were observed against penicillin (88.9%), tetracycline (86.7%) and doxycycline (66.7%). Of the isolates, 71.1% were classified as multidrug-resistant (MDR) organisms, and 60% exhibited a multiple antibiotic resistance index greater than 0.2. PCR analysis revealed the presence of the resistance genes *blaZ* (86.7%), *mecA* (27.3%), *tet*(M) (46.2%), *tet*(K) (35.9%), *tet*(S) (59%), *erm*(B) (51.9%), and *erm*(C) (59.3%). A total of 44 isolates were biofilm producers: 46.7% were weak producers, 46.7% were moderate producers, and 4.4% were strong producers. These findings highlight a significant public health concern, emphasizing the need for stringent hygiene practices and continuous monitoring to limit the spread of resistant pathogens through the food chain.

## 1. Introduction

*Staphylococcus aureus* (*S. aureus*) is a commensal and opportunistic bacterium with a broad host range capable of causing a wide range of infections, from minor skin and soft tissue infections to severe, life-threatening conditions such as sepsis and toxic shock syndrome [1]. Furthermore, it is a significant foodborne pathogen, recognized as one of the mean causes of food poisoning. Globally, it is classified as the third leading causative agent of foodborne illnesses, following Salmonella and Vibrio parahaemolyticus [2,3]. One of the most critical public health concerns in recent decades is the rapid emergence and spread of antimicrobial resistant (AMR) bacteria. In poultry farming, antibiotics are routinely used not only for the treatment of infections but also as growth promoters and for prophylactic purposes [4]. The overuse and often inappropriate use of antimicrobials in the poultry sector exerts strong selective pressure that contributes to the emergence of resistant bacterial strains. These AMR strains can be transmitted to humans through the food chain, primarily via zoonotic pathogens many of which are foodborne bacteria present in contaminated animal-derived products such as meat and meat products, thereby posing a significant risk to public health [5,6,7]. Among the key characteristics of *S. aureus* is its remarkable ability to adapt to varying environmental conditions and rapidly acquire resistance to multiple antibiotic agents [3,8]. In recent years, multidrug-resistant *S. aureus* strains have been increasingly reported in foodborne outbreaks and isolated from a wide range of food products, particularly chicken meat [3,9]. Methicillin-resistant *S. aureus* (MRSA) has gained significant attention due to its resistance to β-lactam antibiotics and its ability to acquire resistance to additional classes of antimicrobial agents, making infections caused by this pathogen difficult to treat [9,10,11]. The zoonotic transmission of MRSA from food-producing animals to humans has been well-documented and represents a significant public health concern [1]. In addition, *S. aureus* has the ability to form biofilms which can develop on a variety of biotic and abiotic surfaces such as human tissues, medical devices, food products, and food processing equipment [8,12]. Biofilm formation begins with attachment of bacterial cells to the surface, followed by the phases of accumulation, maturation, and eventual detachment, which facilitate the dissemination of staphylococci [13]. The biofilm matrix protects bacteria from cleaning and disinfection procedures, impedes the penetration of antimicrobial agents, and enables the bacteria to escape host immune responses, thereby allowing the bacteria to persist in the environment [14]. In meat processing facilities, the formation of biofilms on contaminated surfaces creates a persistent source of contamination posing a significant challenge to the food industry [15]. Slaughterhouses are considered a primary point of contamination in the meat production chain, and chicken meat is increasingly recognized as a reservoir for antimicrobial-resistant bacteria. This study was conducted to isolate *S. aureus* from raw chicken meat collected at slaughterhouses, evaluate the antimicrobial susceptibility profiles of the isolates, detect selected resistance genes, and assess their biofilm-forming ability.

## 2. Materials and Methods

### 2.1. Sample Collection

A total of 130 chicken meat samples were randomly collected from different slaughterhouses located in Setif Province, Eastern Algeria, between September 2021 and January 2023. The samples were aseptically sampled, placed in sterile sampling bags and immediately transported to the Laboratory of Applied Microbiology, University of Setif 1, for analysis, under refrigerated conditions using an ice box.

### 2.2. Isolation and Identification of S. aureus

The isolation of *S. aureus* was carried out following the method described by Parvin et al. [16], with modifications. Briefly, ten grams of fresh chicken meat were aseptically cut into small pieces using a sterile tool and transferred into 90 mL of buffered peptone water. The mixture was then incubated at 37 °C for 18 h. Following incubation, a loopful of the enrichment broth was streaked onto Mannitol Salt Agar (Liofilchem, Roseto degli Abruzzi, Italy) and further incubated at 37 °C for 48 h. Two to three putative colonies of *S. aureus* from each plate were streaked on Nutrient Agar (TM-Media, Delhi, India) to obtain pure isolates. Suspected colonies were further identified on the basis of the phenotypic characteristics observed in selective media such as Mannitol Salt Agar and Baird-Parker Agar (VWR, Leuven, Belgium) supplemented with 5% tellurite emulsion (Merck, Darmstadt, Germany), Gram staining, catalase and coagulase tests. Further confirmation was achieved by the API Staph kit (BioMerieux, Marcy-l’Etoile, France). Upon confirmation, one representative *S. aureus* isolate from each positive sample was selected for subsequent analyses. The pure cultures were preserved in 30% glycerol and stored at −20 °C until phenotypic antimicrobial resistance testing and DNA extraction.

### 2.3. Antibiotic Susceptibility Test

All the isolated cultures were examined for their patterns of antimicrobial resistance to 19 antibiotics belonging to 13 classes using Kirby Bauer Disc diffusion method on Mueller Hinton Agar according to the European Committee on Antimicrobial Susceptibility Testing (EUCAST) guidelines [17]. The antibiotics tested were as follows: β-lactams: Penicillins [Amoxicilline/Clavulanic acid (AMC), Penicillin (P), Ampicillin (Amp)] and Cephalosporins [Cefoxitin (FOX), Fosfomycin (Fosfomycin (FF)]; Glycopeptides [Vancomycin (VAN)]; Aminosides [Kanamycin (K), Tobramycin (TOB), Gentamycin (CN)]; Macrolids [Erythromycin (E)]; Lincosamids [Clindamycin (CD)], Tetracyclines [Doxycycline (DO), Tetracycline (Tet)]; Fluoroquinolones [Ciprofloxacin (Cip), Ofloxacin (OFX)]; Rifampicin [Rifampicin (RD)]; Diaminopyrimidines/Sulfamides [Trimethoprim/Sulfamethoxazole (SXT)]; Phenicols [Chloramphenicol (C)]; Fusidic acid (FA). The diameter of inhibition zones surrounding each antibiotic disk was measured, and bacterial susceptibility was subsequently classified as sensitive or resistant in accordance with the breakpoints established by EUCAST. Antimicrobial resistant bacterial isolates were classified into three categories: multidrug-resistant (MDR) bacteria, which are non-susceptible to at least one agent in three or more antimicrobial categories; extensively drug-resistant (XDR) bacteria, which are non-susceptible to at least one agent in all but two or fewer antimicrobial categories; and pan-drug-resistant (PDR) bacteria, which are non-susceptible to all agents in all antimicrobial categories [18]. The multiple antibiotic resistances (MAR) index was determined as described by Anihouvi et al. [19].

### 2.4. DNA Extraction

Genomic DNA from all bacterial isolates was extracted using the boiling method [20]. Briefly, two to three colonies from a pure overnight culture grown on Brain Heart Infusion (BHI) agar were suspended in 300 µL of TE buffer (10 mM Tris-HCl, 1 mM EDTA, pH 8). The suspension was then incubated in a dry bath (HB 120-C, Argolab, Carpi, Italy) at 100 °C for 12 min. After that, the samples were centrifuged at 13,000 rpm for 10 min using a Rotofix 32A centrifuge (Hettich, Milan, Italy). Subsequently, the supernatant was transferred to new tubes and stored at −20 °C for further use. The extracted DNA was assessed for quantity and purity using a NanoDrop ND-1000 spectrophotometer (Thermo Fisher Scientific, Wilmington, DE, USA). DNA samples were then amplified via endpoint PCR using the universal prokaryotic primer pair 388f–518r [21] to verify successful bacterial DNA extraction.

### 2.5. PCR Detection of AR Genes

All isolates exhibiting phenotypic resistance were screened for the presence of AR genes associated with resistance to β-lactams (*blaZ*, *mecA*), tetracycline [(*tet*(K), *tet*(M), *tet*(O), *tet*(S)], erythromycin [(*erm*(A), *erm*(B), *erm*(C)], vancomycin (*vanA*, *vanB*) [22], and aminoglycosides [*aac(6′)-Ie-aph(2″)-Ia*] [23] using end-point PCR. DNA from bacterial references, each carrying one of the AR genes under study, was used as a positive control in each PCR [24]. The amplification reactions were carried out in a final volume of 25 µL each consisting of 0.15 µL of Taq DNA polymerase (5U/µL) (SibEnzyme Ltd., Novosibirsk, Russia), 2 µL of 10X SE-Taq polymerase buffer, 0.5 µL of 10 mM dNTPs, 1 µL of each 10 µM primer, 3 µL of extracted DNA, and sterile, nuclease-free water was added to adjust the final volume. The primers, expected amplification product sizes, and PCR conditions are listed in Table 1. To assess the presence or absence of the tested AR genes in the isolates, five microliters of PCR products were subjected to electrophoresis on a 1.5% (*w*/*v*) agarose gel prepared in 0.5× Tris/Borate/EDTA (TBE) buffer (VWR Chemicals), containing 0.5 μg/mL GelRed^®^ Nucleic Acid Gel Stain (10,000× in water; Biotium, Fremont, CA, USA). Electrophoresis was performed at 100 V for 50 min. The PCR products were visualized under a UV transilluminator and photographed. The expected fragment sizes for each AR gene were verified by comparison with a 100 bp DNA ladder (HyperLadder™ 100 bp, Bioline, London, UK).

### 2.6. Biofilm Formation

*S. aureus* isolates were screened for their ability to form biofilm using 96 microtiter plate method according to [35] with slight modifications. Briefly, two or three colonies from a pure fresh culture were transferred into BHI broth (Condalab, Madrid, Spain) supplemented with 1% glucose and incubated at 37 °C for 24 h. Overnight cultures were diluted 1:100 in sterile medium. 200 μL of each bacterial suspension was inoculated in duplicate into 96-well sterile polystyrene microplates and incubated at 37 °C for 24 h. 200 µL of fresh broth with 1% glucose served as the negative control, and a strain of *S. aureus* 29213 used as the positive control. The bacterial suspensions were aspirated from each well and washed 3 times with 200 μL of phosphate-buffered saline. The wells were air-dried and then stained with 200 μL of 0.1% crystal violet for 15 min. After removing the crystal violet solution (Biochen Chemopharma, Cosne-sur-Loire, France), the wells were rinsed with water, and the plates were kept for drying. 200 uL of ethanol (95%) was added to all the wells, then the optical densities (ODs) of the plates were measured at 570 nm using an ELISA reader. The cut-off for OD was established as follows:

ODc = Average ODnc + 3 × SDnc

where c is the control, nc is the negative control, and SD is the standard deviation.

The isolates were classified into four categories as follows: A: non-biofilm producer (OD ≤ ODc); B: weak biofilm producer (ODc < OD ≤ 2 × ODc); C: moderate biofilm producer (2 × ODc < OD ≤ 4 × ODc); and D: strong biofilm producer (4 × ODc < OD).

### 2.7. Statistics

GraphPad Prism 5.03 was used to design the histograms, while statistical analysis was performed using the SPSS 21.0 statistical software. The Chi-square test and Fisher’s exact two-tailed test were used to assess any significant correlations between biofilm formation, antibiotic resistance patterns, and antibiotic resistance genes. A *p* value < 0.05 was considered statistically significant.

## 3. Results

### 3.1. Prevalence of S. aureus

The present study was conducted to evaluate the prevalence of *Staphylococcus* and its resistance profiles from raw chicken meat samples. This microorganism was present in 34.62% of samples based on phenotypic and biochemical characterization.

### 3.2. Antimicrobial Susceptibility Test

**Figure 1:**
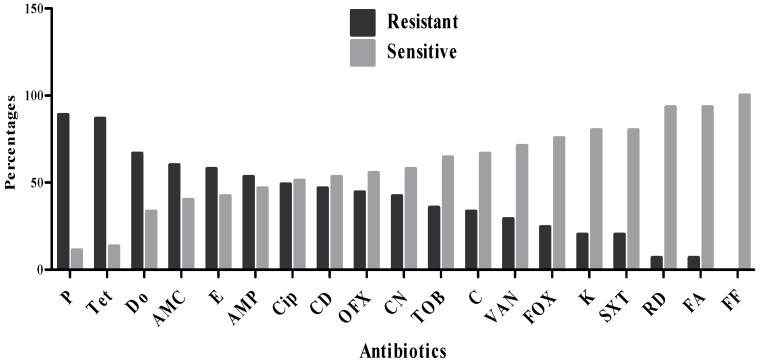
Resistance profile of *S. aureus* strains.

Th antibiogram results revealed varying levels of susceptibility and resistance among the 45 *S. aureus* isolates to the tested antibiotics (Figure 1, Table 2). The highest resistance rates were observed against penicillin (88.9%), tetracycline (86.7%), doxycycline (66.7%), amoxicillin–clavulanic acid (60%), and erythromycin (57.8%). Moderate resistance levels were seen for ampicillin (53.3%), ciprofloxacin (48.9%), clindamycin (46.7%), ofloxacin (44.4%), gentamicin (42.2%), and tobramycin (35.6%). Lower resistance rates were recorded for chloramphenicol (33.3%), vancomycin (28.9%), cefoxitin (24.4%), as well as kanamycin and trimethoprim–sulfamethoxazole (each at 20%). In contrast, the highest susceptibility rates were observed for fosfomycin (100%), rifampicin (93.3%), and fusidic acid (93.3%).

**Figure 2:**
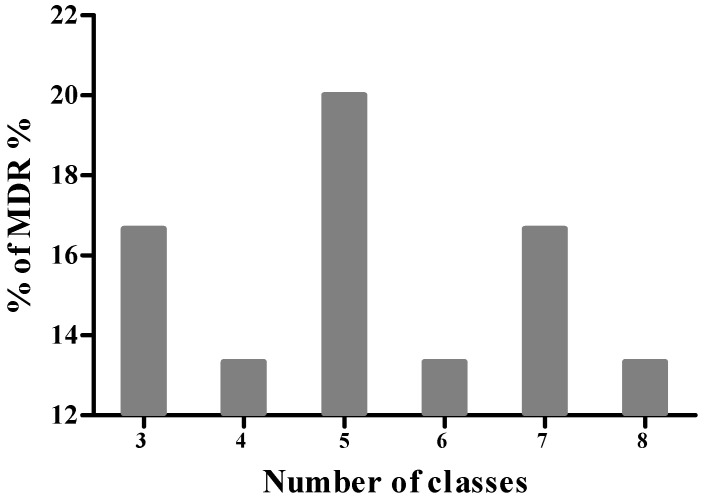
Distribution of multidrug-resistant (MDR) *S. aureus* isolates.

Based on the results of antimicrobial susceptibility testing, none of the isolates were classified as XDR or PDR. However, 71.11% of isolates were identified as MDR, exhibiting resistance to three or more classes of antibiotics. Among the latter isolates, 12.5% were resistant to 3 antibiotic classes, 18.8% to four classes, and 9.4% to five classes (Figure 2). Furthermore, 15.6%, 12.5%, and 18.8% of the isolates demonstrated resistance to six, seven, and eight antibiotic classes, respectively. Notably, 12.5% of the MDR isolates showed resistance to as many as 9 antibiotic classes.

The MAR index was calculated for each isolate (Table 3), ranging from 0.1 to 0.78, with an average value of 0.4. Notably, 88.88% of the isolates exhibited a MAR index greater than 0.2. All 45 isolates exhibited distinct antibiotic resistance patterns, which are presented in Table 3.

### 3.3. Biofilm-Forming Ability

Out of the *S. aureus* isolates examined, only one isolate was identified as a non-biofilm producer (Table 4), whereas the remaining 44 isolates (97.8%) were able to produce biofilm (Table 4). Among the latter microorganisms, 46.7% of isolates exhibited weak biofilm formation, an equal proportion (46.7%) showed moderate biofilm production, and only 2 isolates were classified as strong biofilm producers.

### 3.4. Detection of Resistance Genes

**Figure 3:**
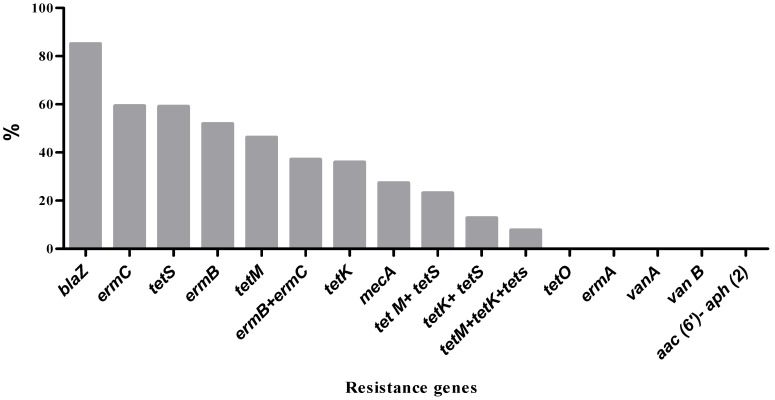
Prevalence of antibiotic resistance genes among *S. aureus* isolates.

Among the isolates phenotypically identified as MRSA based on resistance to cefoxitin, only three isolates were found to harbor the *mecA* resistance gene. The *blaZ* gene, which confers resistance to penicillin, was the most frequently detected, present in approximately 86.7% of penicillin-resistant isolates (Figure 3). Tetracycline-resistant isolates were screened for four *tet* resistance genes. The *tet*(S) gene was the most prevalent, detected in 59% of the isolates, followed by *tet*(M) (46.2%) and *tet*(K) (35.9%). None of the isolates carried the *tet*(O) gene. Some isolates possessed multiple *tet* resistance genes, including combinations such as *tet*(M) + *tet*(S) (23.1%), *tet*(K) + *tet*(S) (12.8%), and *tet*(M) + *tet*(K) + *tet*(S) (7.7%). Regarding erythromycin resistance, three resistance genes were tested. The *erm*(C) gene was identified in 59.3% of resistant isolates, while *erm*(B) was present in 51.9%. The *erm*(A) gene was not detected in any of the isolates. Notably, 37% of the isolates carried both *erm*(B) and *erm*(C) genes. Genes associated with vancomycin resistance (*vanA*, *vanB*) and aminoglycoside resistance *aac(6′)-Ie-aph(2″)-Ia* were not detected in any of the assayed isolates.

### 3.5. Statistical Correlations

#### 3.5.1. Association of Biofilm Formation with Antibiotic Resistance Patterns in *S. aureus* Isolates

The association between biofilm formation and antibiotic resistance patterns was evaluated. Among the non-MDR *S. aureus* isolates, 69.2% were weak biofilm producers, while 30.8% were moderate biofilm producers. In contrast, among MDR isolates, 53.1% were moderate producers, followed by 37.5% weak producers, 6.3% strong producers, and 3.1% non-producers. A statistically significant association was observed between biofilm formation and multidrug-resistant strains at *p* = 0.0001 (Table 5).

#### 3.5.2. Association of Biofilm Formation with Antibiotic Resistance Genes

The distribution of various resistance genes with respect to biofilm formation in *S. aureus* isolates are presented in Table 6. Although there was no statistically significant difference (*p* > 0.05) in the prevalence of resistance genes across the different categories of biofilm-producing isolates, these genes were generally more frequently detected in weak and moderate biofilm producers.

## 4. Discussion

This study was conducted to assess the prevalence and to characterize the phenotypic and genotypic profiles of antibiotic resistance in *S. aureus* isolates from raw chicken meat collected from slaughterhouses in Eastern Algeria. Of the 130 samples analyzed, 34.6% were contaminated with *S. aureus*. These results are closely aligned with those of a previous study conducted in Algeria, which reported that 46.66% of chicken meat samples collected from slaughterhouses were positive for *S. aureus* [36]. Similarly, in Korea, 43.3% of chicken meat samples from slaughterhouses were found to be contaminated with this food-borne pathogen [37]. However, in Morocco, the prevalence was considerably lower, with only 15.92% of chicken meat samples testing positive for *S. aureus* [38]. The presence of *S. aureus* in the meat is often indicative of insufficient control measures and inadequate personal hygiene practices [39]. These differences in the prevalence of *S. aureus* may be attributed to various factors, including study design, sampling techniques, sample size, identification methods, and slaughterhouse equipment used (e.g., knives, slicing machines, wiping cloths) [39,40]. The management of staphylococcal infection relies on antimicrobial therapy, which often fails because of the strong resistance of the bacteria to certain drugs [16]. In the present study, among penicillins, the highest resistance was observed against penicillin G (88.9%), followed by amoxicillin–clavulanic acid (60%) and ampicillin (53.3%). Similar findings have been reported in previous studies, where *S. aureus* isolates from chicken meat exhibited high levels of resistance to penicillin G [41,42,43]. Other authors [38,42] reported that 100% of *S. aureus* isolates were resistant to ampicillin. Consistent with our findings, Islam and colleagues [44] reported a resistance rate of 61.54% to amoxicillin–clavulanic acid. *S. aureus* is recognized for its notable resistance to the penicillin class of antimicrobials, which has been documented in Gram-positive bacteria since 1940s. Cefoxitin is used as a reliable indicator for the detection of methicillin resistance [6]. Based on the results of resistance to cefoxitin, 24.4% of *S. aureus* isolates were identified as MRSA. A comparable prevalence of MRSA in chicken meat has been reported in previous studies, including 19.4% in Benin and 20.8% in South Africa [19,45]. In contrast, a significantly higher prevalence rate of 63% and 42,31% has been reported in Pakistan and Bangladesh, respectively [6,44]. In the tetracycline class, a notably high resistance rate was observed against tetracycline (86.7%), followed by doxycycline (66.7%). These findings are consistent with those of [38,41] who reported tetracycline resistance in *S. aureus* isolated from fresh chicken meat at rates of 80.4% and 100%, respectively. Furthermore, our results align with those of [46] regarding resistance to doxycycline. The resistance to tetracycline was not surprising, as it is extensively used in poultry farms due to its low price and minimal side effects to promote growth [47]. The observed resistance to erythromycin (57.8%) and clindamycin (46.7%) corroborates with the findings of [48]. Among the fluoroquinolones tested in this study, moderate levels of resistance were observed against ciprofloxacin (48.4%) and ofloxacin (44.4%). Compared to the findings of other studies conducted in Algeria, both antibiotics showed lower resistance rates, with 14.28% of *S. aureus* isolates from chicken meat resistant to ofloxacin [49], and 13% and 19% of isolates from artisanal sausage resistant to ciprofloxacin and ofloxacin, respectively [50]. Our results indicated that resistance to gentamicin (42.2%) was notably high among the aminoglycoside class of antibiotics, contrasting with earlier studies conducted in the same country, which reported lower resistance rates to this antibiotic [51,52]. Despite the official prohibition of chloramphenicol by the Algerian government, 33% of the isolates demonstrated resistance to this antibiotic. This observation may be explained by the illicit use of chloramphenicol in poultry farming [50]. Fortunately, the isolates in this study demonstrated high susceptibility to fosfomycin, rifampicin, fusidic acid, trimethoprim–sulfamethoxazole, and vancomycin. The high sensitivity observed against these antibiotics may be due to their limited or absent application in veterinary medicine, which reduces the selective pressure for resistance development. However, the variation in antibiotic resistance rates between and within countries may be attributed to differences in animal husbandry practices, sanitary conditions, and the types of antimicrobials used on farms [53]. All *S. aureus* isolates were resistant to at least one of the antibiotics tested. Furthermore, 71.11% of the isolates exhibited multidrug resistance. These findings are consistent with those reported by Li and colleagues [9] and Nacer and colleagues [38], who observed that 99.7% and 100% of *S. aureus* isolates, respectively, were resistant to at least one antibiotic. However, both studies reported higher rates of multidrug resistance compared to the findings of our study. The MAR index serves as a valuable tool for evaluating the potential health risks associated with antimicrobial resistance in bacteria and for determining whether bacterial isolates originate from environments characterized by low (MAR index ≤ 0.2) or high (MAR index > 0.2) antibiotic usage. The MAR index is calculated as the ratio of the number of antibiotics to which an organism exhibits resistance to the total number of antibiotics tested [54]. In this study, MAR index values exceeding the threshold of 0.2 were observed in 88.88% of the isolates, indicating that these bacteria likely originated from high-risk sources where multiple antibiotics are frequently used, potentially due to contamination of human or animal origin [19]. MRSA has emerged as a significant food safety concern and represents a major challenge in healthcare. The confirmation of MRSA is based on the detection of the *mecA* gene, a highly conserved genetic determinant among staphylococcal species, which serves as a reliable biomarker for methicillin resistance [6,16]. In this study, only three of the phenotypically cefoxitin-resistant isolates were found to harbor the *mecA* resistance gene. The isolates that lack the *mecA* gene may exhibit resistance due to the presence of the *mecC* gene, which was not investigated in our study [55]. In close agreement with our findings, previous research conducted in Algeria reported that, among 30 phenotypically identified MRSA isolates from various food sources, only 3 strains carried the *mecA* gene [52]. However, in another study, approximately half of the MRSA isolates recovered from food sources were found to harbor the *mecA* gene [56]. Resistance to penicillin and its analogues in *S. aureus* is primarily mediated by the production of β-lactamase, encoded by the *blaZ* gene [57,58]. This resistance gene was frequently detected among the penicillin-resistant isolates in our study, which is in concordance with the findings of Igbinosa et al. [15]. The resistance to tetracycline in *S. aureus* is mainly due to two mechanisms: active efflux mediated by *tet*(K) and ribosomal protection conferred by *tet*(M), *tet*(O), and *tet*(S) genes [55,59]. The most frequently detected genes associated with tetracycline resistance in other studies were *tet*(K) and *tet*(M), which is consistent with our findings [15,43,60]. However, our isolates also harbored the *tet*(S) gene, which was the most prevalent. The absence of the *tet*(O) gene in all tested isolates aligns with the findings reported by Zehra and colleagues [10].

The erythromycin ribosomal methylase (*erm*) genes confer resistance not only to macrolides but also to lincosamides and streptogramin B by encoding methyltransferases that methylate the 23S rRNA of the 50S ribosomal subunit [61,62]. In contrast to the findings of Chouaib et al. [52] which identified only the *erm*(C) gene, the erythromycin-resistant isolates in the present study harbored *erm*(C) and/or *erm*(B). A previous study conducted in Algeria reported that *S. aureus* isolates from clinical samples carried *erm*(C) or *erm*(A), the latter of which was completely absent in our isolates [63]. One of the primary mechanisms by which staphylococci develop resistance to aminoglycosides is the inactivation of the antibiotic by aminoglycoside-modifying enzymes (AMEs). These enzymes are encoded by various resistance genes, such as *aac(6′)-Ie-aph(2″)-Ia*, which was absent in all aminoglycoside-resistant isolates examined in our study. The observed resistance may therefore be attributed to the presence of other AME genes not investigated in this study or to reduced permeability of the bacterial cell wall [55,64,65]. Vancomycin is considered a last-resort antibiotic, primarily reserved for the treatment of severe infections caused by Gram-positive bacteria, particularly multidrug-resistant organisms such as MRSA. However, *S. aureus* has developed resistance mechanisms against vancomycin, most notably through the acquisition of *van* gene clusters, especially the *vanA* and *vanB* genes [66,67]. In this study, none of the vancomycin-resistant isolates harbored the *vanA* or *vanB* genes, which contrasts with the findings of previous research. Elshebrawy and colleagues [66] reported the presence of the *vanA* gene in *S. aureus* isolates obtained from chicken carcasses, sandwiches, and buffalo milk, while the *vanB* gene in *S. aureus* isolates from foods of animal origin. *S. aureus* may employ alternative mechanisms to resist vancomycin, such as increasing the thickness of the bacterial cell wall, which limits the antibiotic’s access to its target site [67,68]. One of the key virulence factors of *S. aureus* is its ability to form a complex extracellular polymeric biofilm, which enables the bacteria to survive under various hostile environmental conditions. The biofilm offers protection against standard disinfection procedures, including detergents and sanitizing agents, as well as environmental stressors, host immune responses, and antimicrobial treatments. As a result, biofilm formation plays a crucial role in persistent contamination and chronic infections across clinical and environmental settings [46,69,70,71]. In the present study, the biofilm forming test revealed that 97.8% of the isolates were able to form biofilms. Among these, 4.4% were classified as strong biofilm producers, 46.7% as moderate producers, and 46.7% as weak or low-level producers. These findings are in line with previous studies from Algeria, where all *S. aureus* isolates recovered from various food sources demonstrated biofilm-forming capacity using the same Method [11,71]. Similarly, in Egypt, 60% of MRSA isolates from raw chicken meat were reported as biofilm producers, with nearly all showing strong biofilm-forming potential [46]. In Nigeria, Igbinosa et al. [15] also reported that 76.36% of MRSA isolates obtained from poultry meat were capable of biofilm production.

## 5. Conclusions

This study reveals a high prevalence of multidrug-resistant (*MDR*) *Staphylococcus aureus* with strong biofilm-forming ability in raw chicken meat from slaughterhouses in the Setif province. The detection of *MRSA* isolates, multiple resistance genes, and elevated MAR index values indicates that poultry meat constitutes an important reservoir of antibiotic-resistant *S. aureus*, representing a serious threat to public health. These results emphasize the urgent necessity for comprehensive control actions—including rigorous hygiene measures in slaughterhouses, stricter regulation of antibiotic use in poultry production (particularly for critically important antimicrobials), and the establishment of a national antimicrobial resistance surveillance system. Moreover, the integration of alternative strategies such as probiotics, bacteriophage therapy, and vaccination should be prioritized. Implementing evidence-based interventions, modeled after successful programs in the EU and other countries, is essential to safeguard public health and strengthen food safety in Algeria.

## Figures and Tables

**Table 1:** Antimicrobial resistance genes targets, primers, and PCR conditions.

Target Gene	Primer Sequence (5′-3′)	Ta (°C)	Product Size (bp)	PCR Programs	GenBank Accession Number or Available References
*tet*(M)	1-ACCCGTATACTATTTCATGCACT 2-CCTTCCATAACCGCATTTTG	48	1115	●	[25]
*tet*(W)	1-GAGAGCCTGCTATATGCCAGC 2-GGGCGTATCCACAATGTTAAC	62	168	●●	[26]
*tet*(O)	1-AACTTAGGCATTCTGGCTCAC 2-TCCCACTGTTCCATATGCTCA	62	519	●●	[27]
*tet*(S)	1-CAATACGAGAGCCGGGTTTC ^a^ 2-ACAACGGGCTGGAATTTCAC ^a^	60	382	●	AM039486;
*tet*(K)	1-TCGATAGGAACAGCAGTA 2-CAGCAGATCCTACTCCTT	55	169	●●●	[28]
*erm*(A)	1-CAGGAAAAGGACATTTTACCAA 2-CTTCGATAGTTTATTAATATTAGT	50	572	●●	[25]
*erm*(B)	1-GAAAAGGTACTCAACCAAATA 2-AGTAACGGTACTTAAATTGTTTAC	54	639	●●	[29]
*erm*(C)	1-TCAAAACATAATATAGATAAA 2-GCTAATATTGTTTAAATCGTCAAT	50	642	●●	[29]
*vanA*	1-GGGAAAACGACAATTGC 2-GTACAATGCGGCCGTTA	58	732	●●●	[30]
*vanB*	1-ATGGGAAGCCGACAGTC 2-GATTTCGTTCCTCGACC	58	635	●●●	[31,32]
*mecA*	1-GGGATCATAGCGTCATTATTG 2-AGTTCTGCAGTACCGGATTTGC	58	1429	●●●	[25,33,34]
*blaZ* *aac(6′)-Ie-aph(2″)-Ia*	1-ACTTCAACACCTGCTGCTTTC 2-TAGGTTCAGATTGGCCCTTAG 1-GAGCAATAAGGGCATACCAAAAATC 2-CCGTGCATTTGTCTTAAAAAACTGG	58 60	240 505	●●● ●●●●	

Ta: annealing temprature. PCR Programs: ● Initial denaturation 95 °C 10 min., 35 cycles at 94 °C 1 min., Ta 1 min, 72 °C 2 min. Final extension 72 °C 7 min. ●● Initial denaturation 95 °C 10 min., 35 cycles at 94 °C 1 min., Ta 1 min., 72 °C 1 min. Final extension 72 °C 7 min. ●●● Initial denaturation 95 °C 10 min., 35 cycles at 94 °C 30 s, Ta 30 s, 72 °C 30 s. Final extension 72 °C 7 min. ●●●● Initial denaturation 94 °C 5 min., 35 cycles at 94 °C 30 s, Ta 30 s., 72 °C 45 s. Final extension 72 °C 6 min. a: a primer designed for this study.

**Table 2:** Prevalence of antibiotic resistance among *S. aureus* isolates.

Classes of Antibiotics	Antibiotic	Disk Load (µg)	Number of Bacteria			
			Sensitive	%	Resistant	%
Penicillins	Amixicillin/clavulanic acid	30	18	40	27	60
	Penicillin	10	5	11.1	40	88.9
	Ampicillin	10	21	46.7	24	53.3
Cephems	Cefoxitin	30	34	75.6	11	24.4
	Fosfomycin	200	45	100	0	0
Glycopeptides	Vancomycin	30	32	71.1	13	28.9
Amioglycosides	Kanamycin	30	36	80	9	20
	Tobramycin	10	29	64.4	16	35.6
	Gentamycin	10	26	57.8	19	42.2
Marcrolides	Erythromycin	15	19	42.2	26	57.8
Lincosamides	Clindamycin	2	24	53.3	21	46,7
Teteacyclines	Doxycycline	30	15	33.3	30	66.7
	Tetracycline	30	6	13.3	39	86.7
Fluoroquinolones	Ciprofloxacin	5	23	51.1	22	48.9
	Olfoxacin	5	25	55.6	20	44.4
Ansamycins	Rifampicin	30	42	93.3	3	6.7
Folate Pathway Inhibitors	Trimethorpim sulfamethoxazole	25	36	80	9	20
Phenicols	Chloromphenicol	30	30	66.7	15	33.3
Fusidan	Fusidic acid	10	42	93.3	3	6.7

**Table 3:** Antibiotic resistance profiles and MAR index of *S. aureus* isolates.

Antibiotic Resistance Patterns	Number of Antibiotics	Number of Classes	MAR Index
P, DO, TET, C	9	3	0.21
E, TET, CIP, OFX, RD	5	4	0.26
AMC, P, VAN, TOB, GEN, E, CD, DO, TET, CIP, OFX, SXT, C	13	8	0.68
TET, FA	2	2	0.1
P, FOX, TET	3	2	0.15
E, CD, DO, TET	4	2	0.21
P, E, CD, DO, TET	5	3	0.26
E, CD, DO, TET	4	2	0.21
P, TET	2	2	0.1
P, AMP, DO, TET	4	2	0.21
AMC, P, AMP, K, E, CD, DO, TET, CIP, OFX, RD, SXT, C	13	8	0.68
AMC, P, AMP, FOX, GEN, TET	6	3	0.31
P, K, Tob, GN, E, CD, DO, Tet, OFX, RD, SXT	11	7	0.57
AMC, P, AMP, TET	4	2	0.21
AMC, P, AMP, TET	4	2	0.21
TET, CIP, OFX	3	2	0.15
AMC, P, AMP, E, CD, DO, TET, CIP, OFX, C	10	5	0.52
AMC, P, AMP, TOB, E, CD, DO, TET, CIP, OFX, SXT, C	12	7	0.63
AMC, P, AMP, DO, TET	5	2	0.26
AMC, P, TOB, GEN, E, CD, DO, TET, CIP, OFX, C	11	6	0.57
AMC, P, AMP, VAN, TOB, E, CD, DO, TET, CIP, OFX, SXT, C	13	8	0.68
AMC, P, AMP, VAN, TOB, E, CD, DO, TET, CIP, OFX, C	12	7	0.63
AMC, P, AMP, GEN, E, CD, DO, TET, CIP, OFX, C	11	6	0.57
AMC, P, AMP, DO, TET, CIP	6	3	0.31
AMC, P, AMP, FOX, VAN	5	2	0.26
AMC, P, AMP, VAN, TOB, GN, DO, TET, CIP	9	5	0.47
AMC, P, AMP, VAN, K, TOB, GEN, E, CD, DO, TET, CIP, OFX, SXT, C	15	8	0.78
AMC, P, AMP, VAN, E, CD, DO, TET, CIP, OFX, SXT, C	12	7	0.63
AMC, P, AMP, TOB, GEN, E, CD, DO, TET, CIP, OFX, SXT, C	13	7	0.68
AMC, P, AMP, TOB, GEN, E, CD, DO, TET, CIP, OFX, C	12	6	0.63
P, VAN, GEN, DO, TET, CIP, OFX	7	5	0.36
P, FOX, TOB, E, CD, DO, TET, C	8	5	0.42
P, E, DO, TET, FA	4	4	0.21
AMC, P	2	1	0.1
AMC, P, AMP, FOX, K, GEN, E, DO, TET, CIP, OFX	11	5	0.57
AMC, P, VAN, GEN, E, CD, DO, TET; CIP, OFX, C	11	7	0.57
AMC, P, AMP, FOX, GEN, TET, FA	7	4	0.36
;oµ£AMC, P, AMP, FOX, VAN, K, GEN, E, DO, TET, CIP, OFX	12	6	0.63
AMC, P, AMP, FOX, VAN, GEN, SXT	7	4	0.63
AMC, P, AMP, FOX, CEN	5	2	0.26
VAN, K, TOB, E, CD, DO, TET, CIP, OFX	9	5	0.47
FOX, K, TOB, GEN, E, SXT	6	4	0.31
FOX, K, TOB, GEN, E, VAN	6	4	0.31
AMX, P, AMP, K, TOB, E, CD, DO, TET	9	4	0.47
P, E, CD, TET	4	4	0.21

AMC: Amoxicilline/Clavulanic acid, AMX: Amoxicillin Clavulanic acid, P: Penicillin, AMP: Ampicillin, FOX: Cefoxitin, VAN: Vancomycin, K: Kanamycin, TOB: Tobramycin, GN: Gentamycin, E: Erythromycin, CD: Clindamycin, DO: Doxycyclin, TET: Tetracyclin, CIP: Ciprofloxacin, OFX: Ofloxacin, RD: Rifampicin, SXT: Trimethoprim sulfamethoxazole, C: Chloromphenicol, FA: Fusidic acid.

**Table 4:** Distribution of *S. aureus* non-producer and producer biofilm.

Degree of Biofilm Formation	Number of Isolates	%
Non-producer	1	2.2
Weak	21	46.7
Moderate	21	46.7
Strong	2	4.4

**Table 5:** Biofilm formation among non-MDR and MDR *S. aureus*.

Degree of Biofilm Formation	Non MDR	MDR
Non Poducer	0	1 (3.1%)
Weak Producer	9 (69.2%)	12 (37.5%)
Moderate Producer	4 (30.8%)	17 (53.1%)
Strong Producer	0	2 (6.3%)
*p* value	0.267	0.0001

**Table 6:** Distribution of resistance genes based on categories of biofilm formation.

Resistance Genes	Non-Producer	Weak Producer	Moderate Producer	Strong Producer	*p*-Value
*macA*	0	1 (33.3%)	2 (66.7%)	0	0.833
*blaZ*	1 (2.9%)	16 (47.1%)	15 (44.1%)	2 (5.9%)	0.674
*tetM*	0	8 (44.4%)	8 (44.4%)	2 (11.1%)	0.266
*tetK*	1 (7.1%)	4 (28.6%)	8 (57.1%)	1 (7.1%)	0.131
*tetS*	0	11 (50%)	10 (45.5%)	1 (4.5%)	0.783
*ermB*	0	6 (42.9%)	6 (42.9%)	2 (14.3%)	0.161
*ermC*	0	9 (56.3%)	7 (43.8%)	0	0.616

## Data Availability

All data generated or analyzed in this study are included in the published article, and additional raw data can be obtained from the corresponding authors upon reasonable request.
